# An Epigenomic Meta-Analysis of Differentially Methylated Sites in Pre- and Post-Metabolic/Bariatric Surgery Adult Female Patients

**DOI:** 10.3390/epigenomes9030032

**Published:** 2025-08-29

**Authors:** Agnieszka Lovett, Graham A. Hitman, Georgios K. Dimitriadis, Alice M. Murphy, Gyanendra Tripathi, Aparna Duggirala

**Affiliations:** 1 OMICS Facility, Biomedical and Clinical Science Research Theme, School of Science, University of Derby, Derby DE22 1GB, UK; a.wisniewska@derby.ac.uk; 2 Centre for Genomics and Child Health, Blizard Institute, Faculty of Medicine and Dentistry, Queen Mary University of London, 4 Newark St., London E1 2AT, UK; g.a.hitman@qmul.ac.uk; 3 Faculty of Life Sciences and Medicine, School of Cardiovascular & Metabolic Medicine and Sciences, Obesity, Type 2 Diabetes and Immunometabolism Research Group, King’s College London, London SE5 9RS, UK; georgios.dimitriadis@kcl.ac.uk; 4 Metabolic Health Theme, Centre for Systems Health and Integrated Metabolic Research, Department of Biosciences, School of Science and Technology, Nottingham Trent University, Clifton, Nottingham NG11 8NS, UK; alice.murphy@ntu.ac.uk

**Keywords:** obesity, epigenetic signatures, methylation, epigenetic, metabolic surgery, bariatric surgery, weight reduction, CpG

## Abstract

Background/Objectives: Metabolic/bariatric surgery is currently the most successful treatment for patients with obesity; however, a fifth of patients undergoing surgery may not lose enough weight to be considered successful. Recent studies have shown that bariatric/metabolic surgery alters the epigenome and may explain postoperative improvements in metabolic health. The primary objective is to consolidate published differentially methylated CpG sites in pre- and post-metabolic/bariatric surgery female patients and associate them with the respective genes and pathways. Methods: This systematic review adhered to the PRISMA-P guidelines and was registered with the PROSPERO (CRD42023421852). Following an initial screening of 541 studies using COVIDENCE, six studies were selected, comprising three epigenome-wide association studies (EWAS) and three candidate gene methylation studies. The published studies collected DNA samples from female patients with obesity before and after surgery (3 months, 6 months, 9–31 months, and 2 years). KEGG pathway analysis was performed on genes where the extracted CpG sites were located. Results: The meta-analysis showed that 11,456 CpG sites are differentially methylated after a successful weight loss surgery, with 109 sites mapped to genes involved in key metabolic pathways, including FoxO, mTOR, insulin, cAMP, adipocytokine, Toll-like receptor, and PI3K-Akt. Conclusion: The highlighted differentially methylated CpG sites can be further used to predict the molecular signature associated with successful metabolic/bariatric surgery.

## 1. Introduction

Globally, approximately 2 billion adults are overweight, amongst which 650 million have obesity according to the BMI classification [1]. Obesity is a chronic progressive relapsing disease and a global health issue that is associated with a higher risk of developing diseases, such as type 2 diabetes (T2D), atherosclerotic cardiovascular disease (ASCVD), and certain types of malignancy [2]. Lifestyle change and weight reduction strategies are used to support improving quality of life [3].

In the United Kingdom, the National Health Service (NHS) applied the Tiered Care Weight Management Pathway to support successful weight loss and weight maintenance. Tier 1 consists of universal interventions like reinforcement of healthy eating and physical activity, tier 2 covers lifestyle interventions, tier 3 combines services provided by the specialist weight management clinics that consist of therapeutic interventions. Tier 4 consists of metabolic/bariatric surgery supported by a specialist weight management team pre- and post-surgery [4]. Along with 50–70% of excess weight loss (%EWL), 20–30% loss of the patient’s initial weight or post-surgery BMI < 35 kg/m^2^ both insulin resistance and T2D status are reversed through metabolic/bariatric surgery, with significantly greater success rates than pharmacological, exercise and dietary interventions [5,6,7]. However, a fifth of patients undergoing surgery may not lose enough weight to achieve clinically significant downstage or remission of obesity-related complications [5]. Since surgery is advocated for many patients with complex obesity, more information is needed to select the best candidates for surgery and to establish personalized goals for the intervention in each case. Previous studies have, to some extent, identified the molecular markers that correlate with weight reduction and metabolic recovery [8,9]. However, these studies did not compare the patients’ molecular profile before and after a metabolic/bariatric surgery. Investigating the molecular markers and their associated pathways responsible for weight reduction and T2D remission after metabolic surgery would help identify the most suitable candidates, reducing costs for the NHS [5].

Recent studies have shown that bariatric/metabolic surgery alters the epigenome and may explain postoperative improvements in metabolic health [10]. The molecular response and epigenetic patterns are dependent on sex-specific differences and the genetic variation present in the differentially methylated regions [11]. There are a limited number of studies that analyze methylation changes associated with successful metabolic/bariatric surgery in the female population. Highlighting the epigenetic signatures associated with successful metabolic/bariatric surgery could provide valuable insights into the biological mechanisms supporting long-term weight management.

The primary objective of the epigenomic meta-analysis is focused on consolidating the published differentially methylated CpG sites in female patients pre- and post-metabolic/bariatric surgery and associating their responses to the respective genes and pathways. The secondary objective is to identify the specific epigenetic signal that is commonly replicated in all the included studies. This information can be further used to predict the molecular signature associated with successful metabolic/metabolic/bariatric surgery as well as finding new drug targets.

## 2. Results

### 2.1. Summary of Reviewed Studies

**Figure 1:**
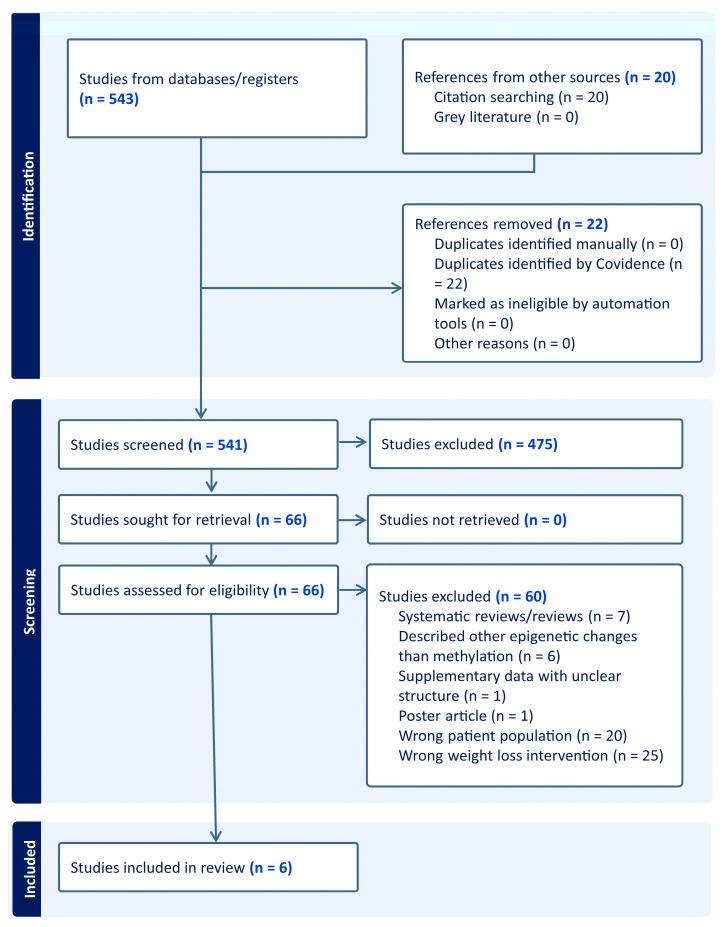
Systematic review flow chart diagram. The PRISMA flow diagram for the systematic review detailing the database searches, the number of studies screened, and studies assessed for eligibility (full-text retrieval).

The search results identified 563 studies from databases, registers, or citation searching, which were uploaded to the Covidence software (www.covidence.org). Duplicates identified by Covidence (*n* = 22) were removed by the software. From the 541 research articles identified during initial screening, 475 articles were excluded during titles and abstract screening. During a full-text screening of the remaining 66 papers, seven were reviews or systematic reviews, six described other epigenetics changes than methylation (e.g., long non-coring RNAs or histone modifications), one had supplementary data with unclear structure, hindering further analysis, 20 wrong patient population (either males or mixed male and female groups), 25 had weight loss interventions different than metabolic/bariatric surgery and one paper was a conference poster article. Thus, a total of six articles were chosen for the review. Preferred Reporting Items for Systematic Reviews and Meta-Analyses (PRISMA-P) was used to present the screening process (Figure 1). The six selected papers were screened for methylation changes to compare pre- and post-intervention data. The publication by Andersson et al. [12] assessed patients 2, 5, and 10 years after metabolic/bariatric surgery; for this review, the extracted results were limited to the first 2 years of the intervention. A wide range of tissues was used in selected publications, including adipocytes, adipose tissue (visceral and subcutaneous), muscle, and blood (including buffy coats and peripheral mononuclear blood cells). Three studies used epigenome-wide DNA methylation analysis (EWAS), and three studies focused on candidate gene methylation analysis. The type of intervention was Roux-en-Y gastric bypass for all papers. Details of each study, including treatment sample size, study design, and duration of data collection, are summarized in Table 1. No papers included a correction for tissue types for instance using the Houseman correction [13] a power calculation, replication studies, and a variety of methods were used to determine statistical significance including the Benjamini–Hochberg or Bonferroni.

### 2.2. Risk of Bias Assessment

**Figure 2:**
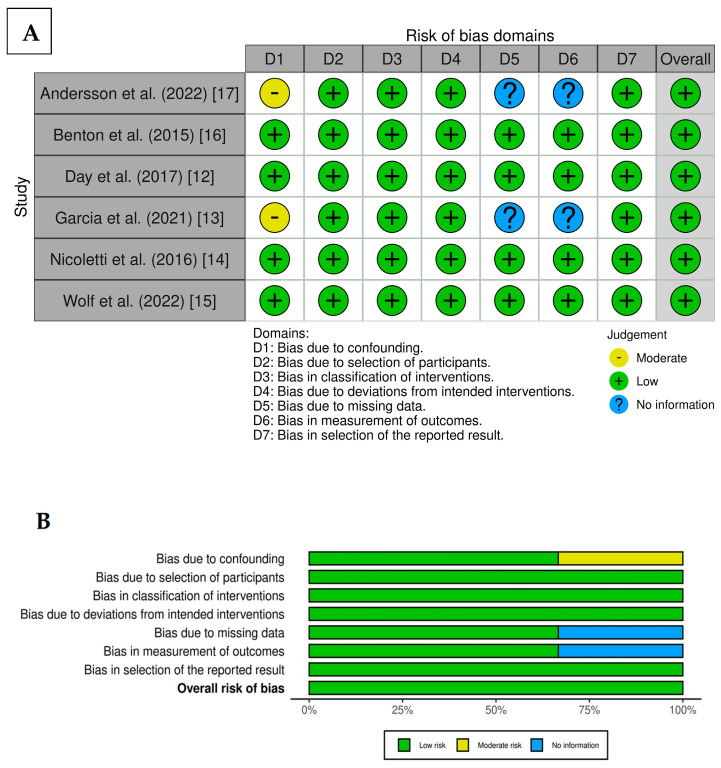
Assessment of risk of bias using (**A**) RoB traffic light plot and (**B**) RoB summary plot generated by robvis [12,13,14,15,16,17].

The assessed risk of bias generated by the robvis ROBINS-I tool was categorized as low for all included publications. A traffic light plot presented in Figure 2A and a summary plot in Figure 2B compile the judgement of risk of bias within included publications. Green color indicates low risk, yellow indicates moderate, and blue indicates unclear risk of bias.

### 2.3. The Analysis of All Methylation Changes Post-Metabolic/Bariatric Surgery

**Figure 3:**
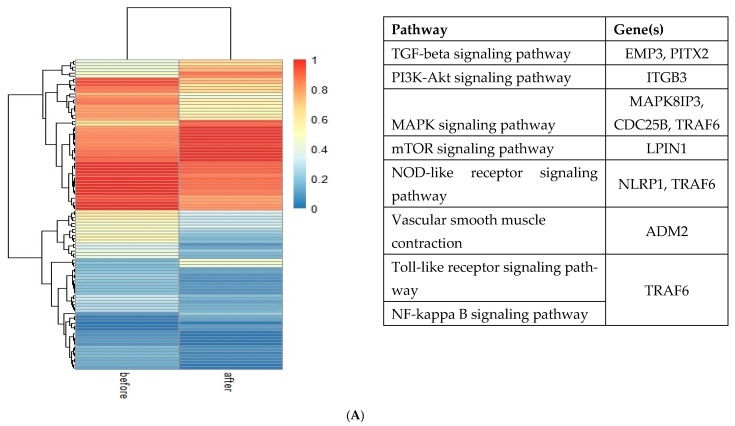

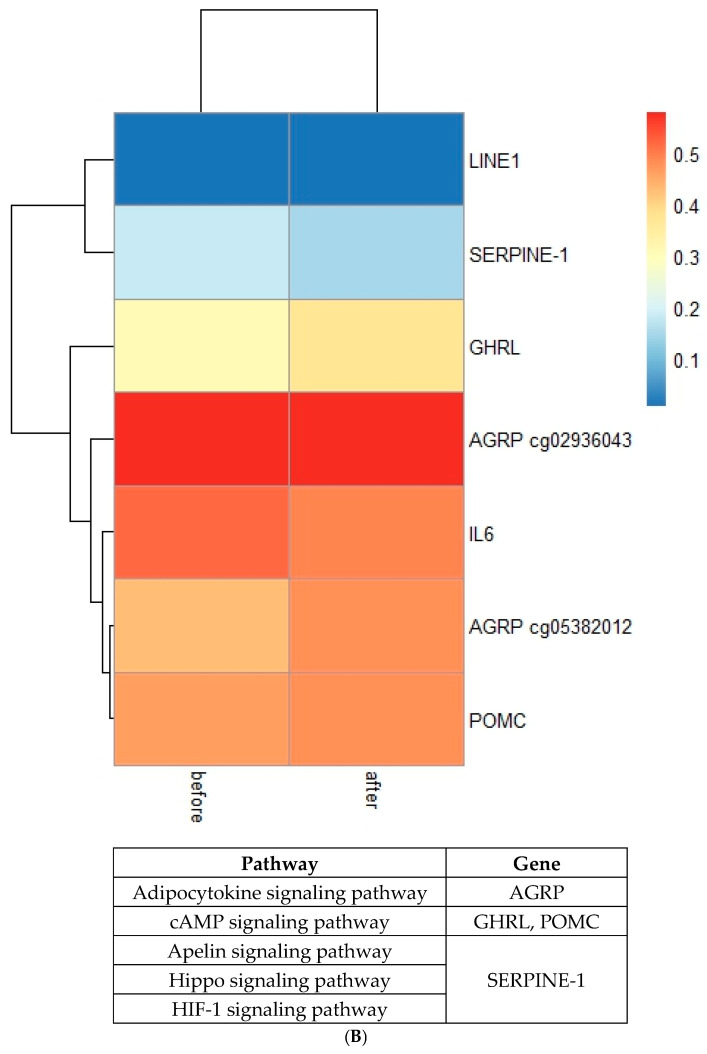

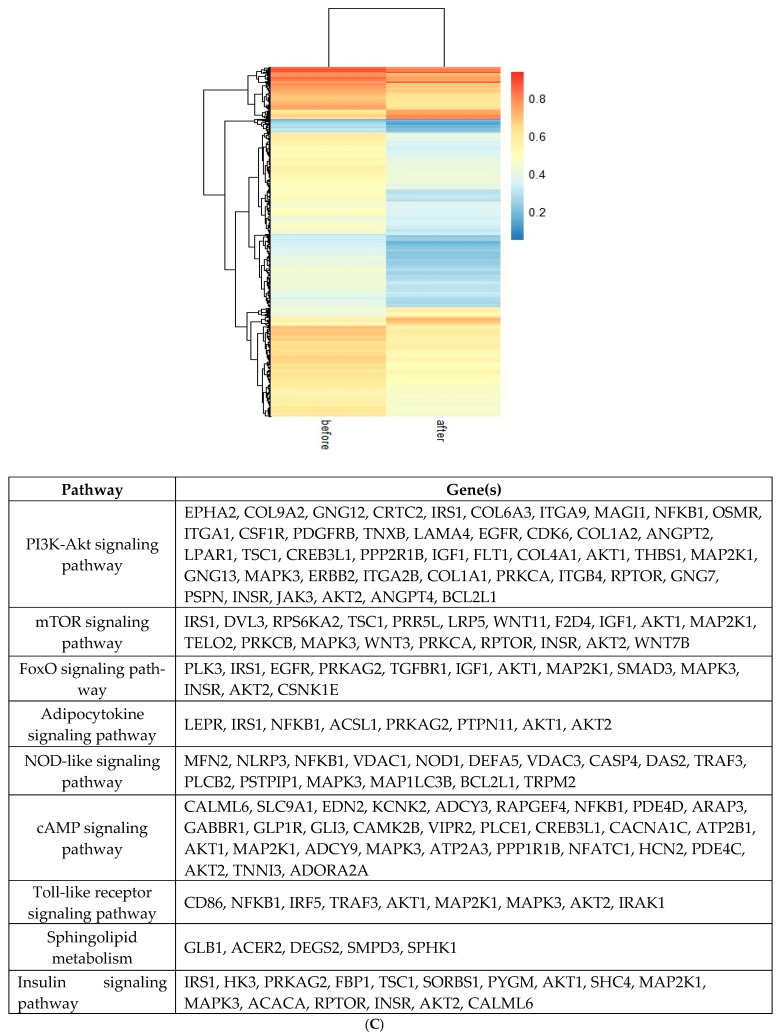

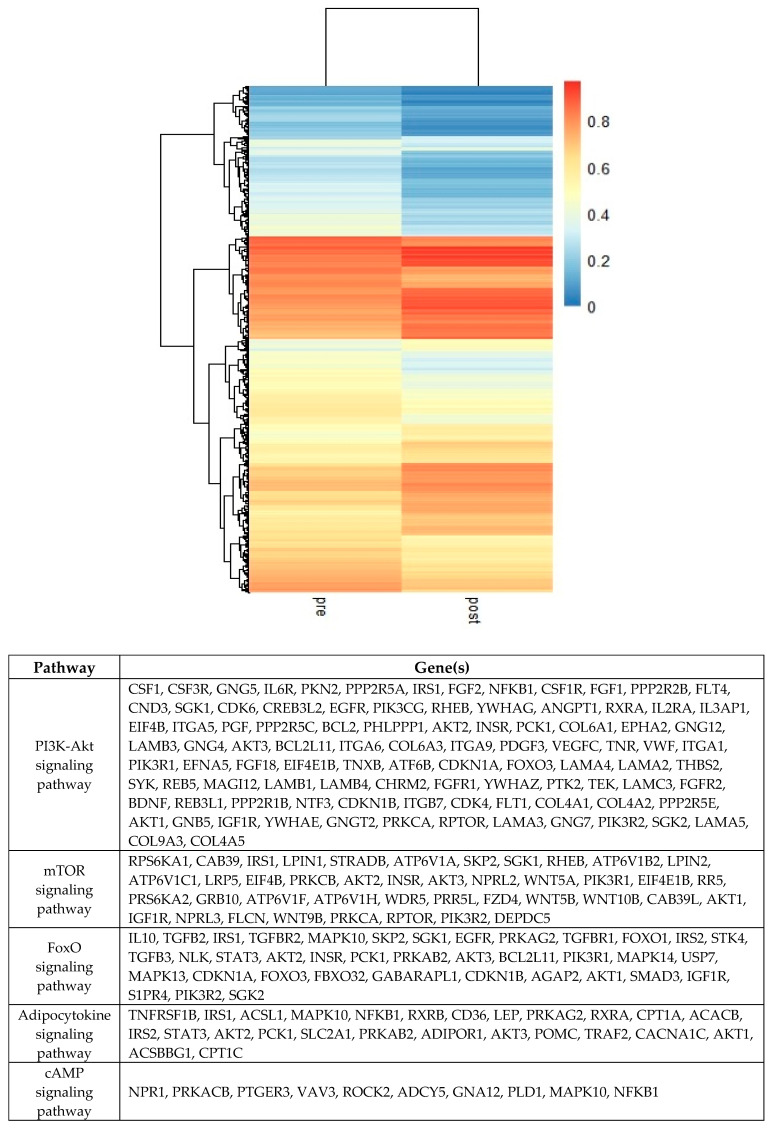

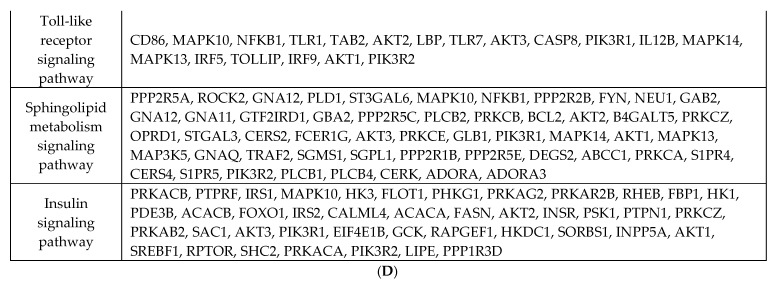
Heat maps of the differentially methylated CpG sites before and (**A**) 3 months, (**B**) 6 months, (**C**) 9–31 months, and (**D**) 2 years after metabolic/bariatric surgery with the summary of selected genes and pathways where the methylation occurred. Data for (**A**) were obtained from two articles [14,15], for (**B**) from two articles [16,17], for (**C**) from one article [18], and for (**D**) from one article [12]. The columns of the heat map represent pre- and post-surgery methylation changes, and the rows represent CpG sites. Tables below each heat map represent all revealed genes (**A**,**B**) or pathways with the highest number of methylation changes and associated genes found in the selected pathways (**C**,**D**).

Data extraction revealed methylation changes in 11,456 sites from six papers. Among all the obtained results, in 6621 CpG sites, the methylation level decreased, in 4834 CpG sites the methylation level increased, and in one, the methylation level did not change post-metabolic/bariatric surgery compared to pre-intervention results. All results were grouped into four time frames and tissue types and presented on the heat maps (Figure 3).

Two studies examined methylation alterations in patients’ muscle tissue three months after the surgery. In the first candidate gene analysis study by Day et al. [14], the analysis of sorbin and SH3 domain-containing 3 gene (*SORBS3*) revealed 30 CpG hypomethylated sites. The results from the epigenome-wide association study (EWAS) in muscle conducted by Garcia et al. [15] showed that 45 CpG sites were hypomethylated and 28 CpG sites were hypermethylated (Figure 3A). Furthermore, the methylation level in one CpG site (cg02936043) within the agouti-related neuropeptide gene (*AGRP*) did not change post-surgery [15].

Nicoletti et al. [16] and Wolf et al. [17] analyzed results from 14 and 24 patients, respectively, using candidate gene analysis from blood samples 6 months after metabolic/bariatric surgery (Figure 3B). Wolf et al. [17] included four CpG sites within three genes: two CpG sites within *AGRP*, one CpG site within ghrelin (*GHRL*), and proopiomelanocortin (POMC). Nicoletti et al. [16] analyzed four CpG sites within long interspersed element-1 *(LINE1*), 5-Hydroxymethylcytosine (5hmC), serpin family E member 1 (*SERPINE-1*), and interleukin 6 (*IL6*). From all analyzed CpG sites, only within the *GHRL* gene methylation increased by 6% [17], whereas for the remaining CpG sites, methylation changes were not significant (below 5%).

Benton et al. [18] analyzed whole genome methylation in omentum and subcutaneous adipose from inter-operative tissue samples from 15 patients at various intervals from 9–31 months (with the mean time of 17.5 months). The majority of the CpG sites in subcutaneous adipose tissue (3281) and omentum (1) were recorded with decreased methylation percentage, whereas in 320 CpG sites in subcutaneous adipose tissue and 14 CpG sites in omentum, the methylation percentage increased over time (Figure 3C). The raw data on the methylation profiling from omentum samples are not available; therefore, the omentum CpG methylation values were not further analyzed. A similar study using subcutaneous adipose tissue samples from 24 patients was conducted by Andersson et al. [12] at the baseline and 2 years post-metabolic/bariatric surgery. They reported that the methylation percentage decreased in 3261 CpG sites, while methylation percentage increased in 4468 regions (Figure 3D).

### 2.4. Differentially Methylated Sites Linked to Metabolic Pathways Were Associated with Successful Weight Loss After Metabolic/Bariatric Surgery

**Figure 4:**
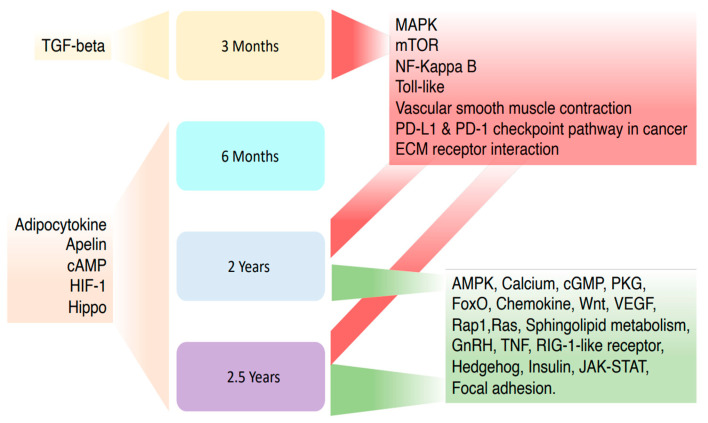
Schematic diagram illustrating the pathways with the highest number of genes revealed during data analysis. The arrow on the top left represents the pathway found only in papers where the intervention took 3 months, the arrow on the bottom left represents pathways associated with genes found in 6 months, 1.5 years, and 2 years, two lines in the bottom right show 9–31 months and 2 years, and three lines at the upper right side show pathways within 3 months, 9–31 months, and 2 years of data collection.

The KEGG pathways analysis of the differentially methylated genes revealed an enrichment in 31 pathways associated with obesity and weight reduction (Supplementary File S1). Selected pathways were grouped based on their appearance in each time frame, as shown in Figure 4.

**Figure 5:**
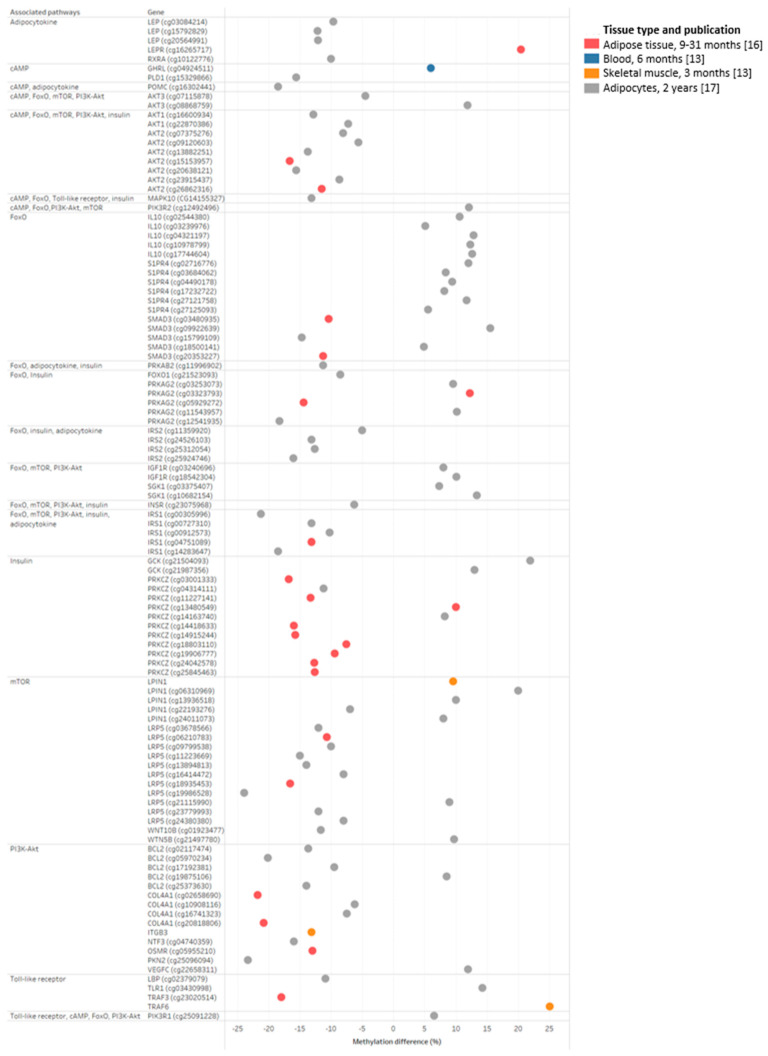
Dot plot of differentially methylated genes after metabolic/bariatric surgery within the selected pathways. The percentage of the methylation difference within the most significant CpG sites was grouped based on the appearance within seven selected pathways (FoxO, Toll-like, mTOR, cAMP, PI3K/Akt, insulin, and adipocytokine). The first column represents the associated pathway(s), the second column represents the most significant CpG sites, and the third column represents the percentage of methylation difference. For each tissue type and the intervention time, there is a different dot color [13,16,17].

After analysis of pooled methylation changes within all time frames, seven pathways forkhead box O (FoxO), Toll-like receptor, mammalian target of rapamycin (mTOR), cyclic AMP (cAMP), phosphatidylinositol 3-kinase/protein kinase B (PI3K-Akt), insulin and adipocytokine] and associated CpG sites within genes linked with obesity or comorbidities development were highlighted (Figure 5).

Eight CpG sites with the most significant methylation changes (>30%) were not associated with any pathway, according to the KEGG database. Five genes [poly(A) binding protein cytoplasmic 1 (*PABPC1*), C2 calcium-dependent domain-containing 4B (*C2CD4B*), MAGE family member L2 (*MAGEL2*), paired like homeodomain 2 (*PITX2*), and SIM bHLH transcription factor 1 (*SIM1*)] were hypomethylated. Those genes were associated with severe obesity and Prader-Willi-like syndrome (*SIM*1) [19], susceptibility to T2D (*C2CD4B*) [20], and susceptibility to obesity with abdominal fat deposition (*MAGEL2*) [21]. The highest hypomethylation levels were revealed within cilia and flagella-associated protein 44 (*CFAP44*, also known as *WDR52*), ArfGAP with dual PH domains 1 (*ADAP1*), DnaJ heat shock protein family (*Hsp40*) member C5 gamma (*DNAJC5G*), and obscurin, cytoskeletal calmodulin, and titin-interacting RhoGEF (*OBSCN*). Those genes are responsible for the regulation of vesicle trafficking and cytoskeletal organization (*ADAP1*) [22], protein folding and the heat shock response (*DNAJC5G*) [23], and muscle organization and integrity (*OBSCN*) [24].

All CpG sites associated with seven selected pathways were next grouped into the specific time frames when the methylation changes were detected (Supplementary File S3). The analysis revealed that from EWAS [15] conducted 3 months post-surgery using muscle tissue, CpG sites within lipin 1 [(*LPIN1*), in mTOR signaling pathway] and TNF receptor-associated factor 6 [(*TRAF6*), in Toll-like receptor signaling pathway] were hypermethylated, whereas one CpG site within integrin subunit beta 3 [(*ITGB3*), in PI3K-Akt signaling pathway] was hypomethylated.

Six months post-surgery, the candidate gene analysis of the blood samples revealed that only one CpG site within *GHRL* associated with the cAMP signaling pathway was hypermethylated [17], whereas other analyzed CpG sites had methylation changes below 5% [16].

In Benton et al. [18], EWAS was conducted on intra-operative adipose tissue samples collected across different time points of 9–31 months post-surgery. The data analysis of subcutaneous tissue revealed that one CpG site within protein kinase AMP-activated non-catalytic subunit gamma 2 [(PRKAG2), in FoxO and insulin signaling pathways] was hypermethylated, whereas 15 CpG sites in FoxO, Toll-like receptor, mTOR, cAMP, PI3K-Akt, insulin, and adipocytokine pathways were hypomethylated.

In the last selected study conducted 2 years post-metabolic/bariatric surgery using subcutaneous adipocytes by Andersson et al. [12], 20 CpG sites within 10 genes were hypermethylated, whereas 28 CpG sites within 21 genes were hypomethylated. The highlighted hypomethylated and hypermethylated CpG sites were found in FoxO, Toll-like receptor, mTOR, cAMP, PI3K-Akt, insulin, and adipocytokine pathways (Figure 5).

### 2.5. The Highlights of Potential Epigenetic Signatures in Three Tissue Types

**Figure 6:**
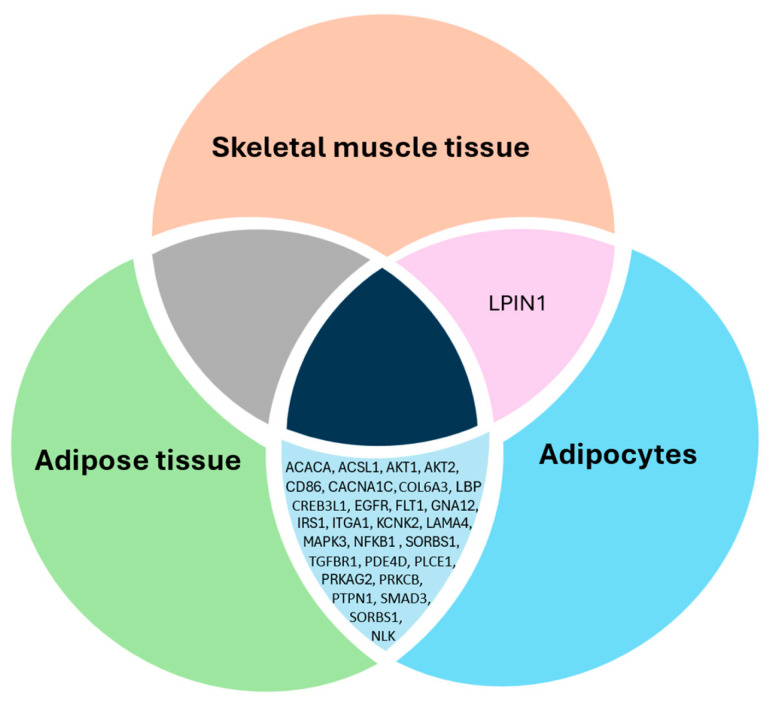
Venn diagram of differentially methylated genes after metabolic/bariatric surgery within three tissue types (adipocytes, adipose tissue, and skeletal muscle tissue) and seven pathways: FoxO, Toll-like, mTOR, cAMP, PI3K/Akt, insulin, and adipocytokine. Three EWAS [12,15,18] were analyzed to determine if there are any common genes that were differentially methylated after successful metabolic/bariatric surgery.

**Figure 7:**
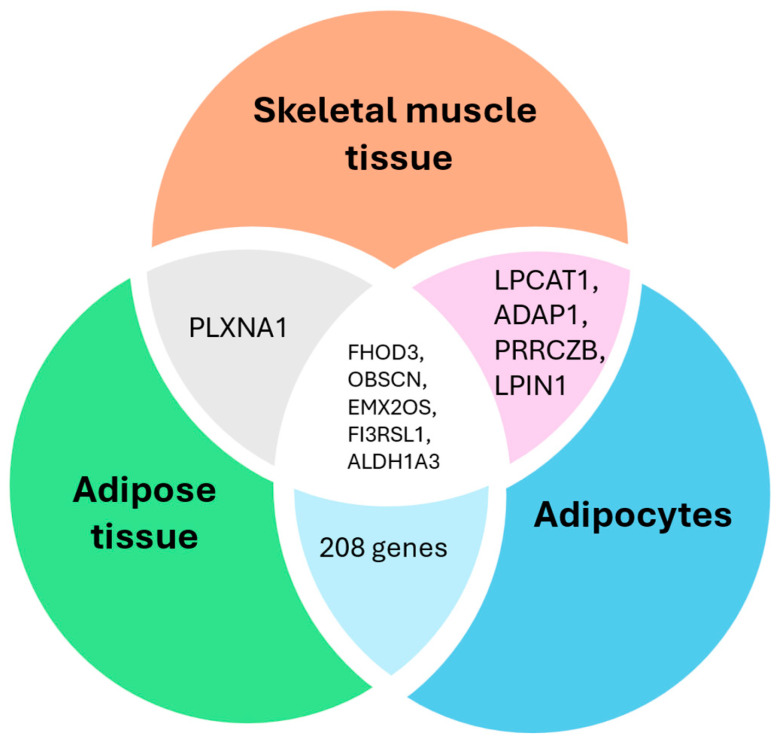
Venn diagram of differentially methylated genes after metabolic/bariatric surgery within three tissue types (adipocytes, adipose tissue, and skeletal muscle tissue) from three EWAS studies [12,15,18]. The analyzed results were not restricted to any specific pathways; the Venn diagram summarizes all available CpG sites from three included papers.

The results from three EWAS [12,16,18] were screened to determine if there are any methylation changes within genes found in three tissue types (adipose tissue, adipocytes, and skeletal muscle tissue). This screening was performed in two steps. Firstly, a biased screening approach was taken where the metabolic pathways related to obesity were selected (FoxO, mTOR, cAMP, PI3K-Akt, insulin, adipocytokine, and Toll-like receptor). The analysis showed that one gene LPIN1 belonging to the mTOR pathway was differentially methylated in skeletal muscle tissue and adipocytes (Figure 6). The EWAS results displayed in Figure 6 from adipose tissue and adipocytes showed that 28 genes were differentially methylated in both tissue types. The details of 28 genes and the associated selected obesity pathway are detailed in Supplementary File S3. There was no gene found within all three tissue types and seven selected pathways. Secondly, an unbiased screening was performed to identify the differentially methylated genes in all three EWAS studies. Five genes are differentially methylated in the three EWAS studies, indicating them as potential epigenetic signals responsible for successful weight loss after a metabolic/bariatric surgery. The five genes shown in the Figure 7 Venn diagram are as follows: formin homology 2 domain-containing 3 (FHOD3), OBSCN, EMX2 opposite strand/antisense RNA (EMX2OS), and aldehyde dehydrogenase 1 family member A3 (ALDH1A3) (Figure 7, Supplementary File S4). However, these functional genes are not associated with any KEGG pathways in the literature.

### 2.6. Genetic Variations Linked with Differentially Methylated CpG Sites

In polygenic obesity, the DNA variants are associated with the development of being overweight and obesity [25]. CpG sites found within seven highlighted pathways were screened to determine the single-nucleotide polymorphisms (SNPs) associated with obesity (full list of revealed SNPs is available in Supplementary File S2). The literature screening revealed six SNPs within four genes, POMC, GHRL, leptin (LEP), and leptin receptor (LEPR), which are detailed in Table 2.

## 3. Discussion

Epigenetic changes observed after metabolic/metabolic/bariatric surgery can influence gene expression involved in metabolism, insulin sensitivity, and adipose tissue function [32]. The current review shows that differentially methylated sites recorded after surgery are associated with weight reduction. The differentially methylated sites belonged to seven molecular pathways which can be linked to the following biological processes: satiety regulation (adipocytokine signaling pathway), adipose tissue hypertrophy and hyperplasia (FoxO, adipocytokine and mTOR signaling pathway), insulin signaling and glucose homeostasis (PI3K-Akt, insulin and cAMP signaling pathway) and low-grade inflammation (Toll-like receptor signaling pathway).

Satiety regulation is a process which controls the appetite and involves the adipocyte signaling pathway. Leptin (LEP) belongs to the adipocytokine signaling pathway and is mainly produced in white adipose tissue [33]. The main functions of LEP are controlling lipolysis and lipogenesis and interacting with nervous and immune systems, suppressing feeding and promoting energy homeostasis after binding to the LEPR to mediate long-term satiety control and controlling lipolysis and lipogenesis [34,35]. The current review showed that after two years of metabolic/bariatric surgery, the CpG sites of LEP and POMC are hypomethylated; thereby potentially increasing the respective gene expression which can be linked with weight reduction due to increased feeling of satiety and decreased hunger [36]. Notably, research indicates that adiponectin levels increase after metabolic/bariatric surgery, which positively affects anthropometric parameters, metabolic profiles, and inflammatory biomarkers [37].

Adipose tissue hypertrophy and hyperplasia involve the increase of adipocyte number and volume, respectively. The adipocyte, FoxO, and mTOR signaling pathways control the biological process of adipose tissue hypertrophy and hyperplasia. Forkhead box O1 (FOXO1) and serum/glucocorticoid-regulated kinase 1 gene (SGK1) play an important role in metabolism and adipocyte differentiation within the FoxO and insulin signaling pathways [38]. Hypermethylation of the SGK1 gene may influence decreased fat tissue development, whereas hypomethylation of FOXO1 may be involved in the conversion of preadipocytes into fat cells. Wnt family member 5B (Wnt5b) and Wnt family member 10B (Wnt10b) are located upstream of the LRP5 gene and belong to the mTOR pathway. The results from the included studies showed that CpG sites within those genes were hypomethylated, which may improve energy balance and body fat loss and decrease the prevalence of T2D [39,40].

The biological processes insulin signaling and glucose homeostasis consist of PI3K-Akt and insulin signaling pathways. According to the studies by Benton et al. [18] and Anderson et al. [12] the gene regions of AKT serine/threonine kinase 1 and 2 (*AKT1* and *AKT2*), mitogen-activated protein kinase 10 (*MAPK10*), protein kinase AMP-activated non-catalytic subunit beta 2 *(PRKAB2), FOXO1, PRKAG2* (one CpG site), insulin receptor substrate 2 (*IRS2*), insulin receptor *(INSR*) and protein kinase C zeta (*PRKCZ*) (two CpG sites) and *LPIN1* (one CpG site) are hypomethylated in post-metabolic/bariatric surgery. The same two studies showed that BCL2 apoptosis regulator (*BCL2*), oncostatin M receptor (*OSMR*), vascular endothelial growth factor C (*VEGFC)*, *PKN2*, and *COL4A1*, which belong to the PI3K-Akt signaling pathway, are hypomethylated in subcutaneous adipose tissue and their function is associated with insulin resistance and lipogenesis [41,42,43,44]. The current epigenomic meta-analysis showed that amongst all the above genes, *LPIN1* methylation change is replicated in adipocytokines and skeletal muscle tissue [16,18]. The available research shows that *LPIN1* gene methylation is decreased in people with obesity, compared to lean individuals [45]. The recently published study by Hinte et al. [46] also replicated similar results in subcutaneous adipose tissue and omentum 2 years after metabolic/bariatric surgery.

Toll-like receptor 1 (*TLR1*) and *TRAF6* were hypermethylated in the current DNA methylation analysis, which can be linked with decreased chronic low-grade inflammation and improved adipose tissue homeostasis [47]. Obesity is linked with chronic low-grade inflammation that can accentuate its common comorbidities [48], whereas metabolic/bariatric surgery improves inflammatory markers in the short term and long term after intervention and is strongly related to change in BMI [49].

The complete analysis of the included EWAS highlighted five genes *FHOD3*, *OBSCN*, *EMX2*, opposite strand/antisense RNA (*EMX2OS*) and aldehyde dehydrogenase 1 family member A3 (*ALDH1A3*) that were not associated with pathways of interest; however, the methylation changes in those genes were found in three tissue types (subcutaneous adipose tissue, adipocytes and skeletal muscle tissue). *ALDH1A3* is involved in the metabolism of retinoids, which are critical regulators of adipocyte differentiation. The ectopic expression of *ALDH1A3* increases intrinsic retinoic acid formation and promotes adipogenesis [50]. In diabetic mice, inhibition of *ALDH1A3* lowered glycaemia and increased insulin secretion [51]. Fewer research papers are available on the ALDH1A3; therefore, further research needs to be undertaken on the *ALDH1A3* gene and its association with weight reduction.

This epigenomic meta-analysis of differentially methylated sites following successful metabolic/bariatric surgery highlights several key pathways that contribute to metabolic health. The key pathways identified are adipocytokine, FoxO, mTOR, insulin, cAMP, PI3K-AKT, and Toll-like receptor signaling pathways. The discussed pathways underline the complex interplay of epigenetic modifications in mediating the metabolic benefits observed in patients with successful weight reduction post-metabolic/bariatric surgery, ultimately contributing to improved overall metabolic health and reduced risk of obesity-related complications. This paper highlights that the differentially methylated sites in the *LPIN1* and *ALDH1A*3 gene regions could be molecular signatures indicating a successful metabolic health recovery after metabolic/bariatric surgery.

### 3.1. Strengths and Limitations

The current epigenomic meta-analysis highlights the methylation signatures that are responsible for successful weight reduction after a metabolic/bariatric surgery. A strength of this review is that it included rigorous inclusion criteria selecting only the data generated on Illumina 450K DNA methylation platform, Illumina HiSeq 2000, or reduced-representation bisulfite sequencing (RRBS). Furthermore, it used short term (3 months and 6 months) and long-term studies (up to 2.5 years), adjusting the possible methylation changes and associated pathways where expression changed significantly (the CpG sites had to meet two criteria: methylation change is more than 5%, and *p* < 0.05) in various time points after metabolic/bariatric surgery [52].

However, this analysis has some limitations. Each publication had a relatively small treatment sample size, ranging between 7 and 24 participants with obesity, which limits the statistical power of the conducted epigenomic meta-analysis. Based on the conducted G*Power (version 3.1.9.7) software calculation [53], an estimated 80% success rate for bariatric surgery was used in the Wilcoxon–Mann–Whitney test and the ratio of participants with successful metabolic/bariatric surgery outcomes to unsuccessful outcomes. Assuming an effect size of 0.8, an alpha level of 0.05, and a desired power of 0.8, the required total sample size for each study should be a minimum of 44 participants. Although the array-based approaches are considered very reliable, potentially novel biomarkers can be missed while using a smaller group of participants, which can result in low statistical power [54]. A significant limitation of the Illumina 450K array is that it only covers approximately 2% of CpG loci in the human genome, which can result in omitting informative sites [55]. Furthermore, the included EWAS have a low replication rate, and validation analyses were not mentioned in the included studies. Only two of the analyzed studies [12,17] included a control group with lean individuals, and none sought replication data sets. For the two longest periods of data collection (9–31 and 24 months), there is only one publication available within the specific follow-up time. Furthermore, 9–31 months is excessively broad and combines diverse responses. Therefore, to overcome this limitation, we analyzed them separately and identified the common pathways associated with each time frame. Another limitation was the different types of tissue used (muscle, blood, subcutaneous adipose tissue, or adipocytes) in the analyzed publications, and that the time frames could not be compared due to dissimilar CpG sites methylation, and revealed associated genes within various pathways that can be enriched in different tissue types. There was no specified list of medications, e.g., obesity management medications that participants may have taken before being offered metabolic/metabolic/bariatric surgery, or any history of concomitant medications. The last limitation is that half of the included publications (*n* = 3) were candidate gene studies, none of which were derived from EWAS studies or internally replicated.

### 3.2. Implications for Future Research

Methylation changes significantly affect gene expression linked with pathways associated with obesity development and energy homeostasis. Due to relatively small sample sizes of each individual study, it will be beneficial to conduct research on larger groups of participants in the discovery analysis, the number of which is informed by power calculations and the additional inclusion of a validation data set in the same tissue type. In addition, future research should potentially focus on the analysis of the methylation changes separately for male and female participants. Epigenetics is essential for understanding metabolic diseases by interacting with genetic and non-genetic factors. Small epigenetic changes can significantly impact disease outcomes due to their heterogeneous nature [56]. Developing epigenetic therapies and technologies requires interdisciplinary collaboration. This field holds great potential for determining response to obesity interventions.

## 4. Materials and Methods

Studies for the review were identified following the Preferred Reporting Items for Systematic Review and Meta-Analysis Protocols (PRISMA-P) and followed methods outlined in The Cochrane Handbook for Systematic Reviews of Interventions [57]. The systematic review was registered with the International Prospective Register of Systematic Reviews PROSPERO (registration number CRD42023421852).

### 4.1. Search Strategy

One author (A.L) conducted systematic searches for eligible studies published up to 12 May 2023 using the Medical Literature Analysis and Retrieval System Online (MEDLINE), SCOPUS (a large, multidisciplinary database), Excerpta Medica (Embase), Cochrane Library, and BioRxiv (title + abstract + keywords). Search terms used for papers screening were: obesity and metabolic/bariatric surgery and epigenetic or DNA methylation. To support a comprehensive literature search, a reference list of relevant articles was screened.

### 4.2. Inclusion and Exclusion Criteria

The publication selection eligibility criteria were established using the population, intervention, comparison, outcome, and study (PICOS) framework (Table 3). The publication selection was based on the following inclusion criteria: female non-pregnant adults (18 years or older) with obesity. The DNA methylation in obese individuals often differs between males and females and is observed in various tissues, including adipose tissue, blood, and muscle. Furthermore, from all conducted bariatric surgeries, 80% of patients are females; therefore, only females were included in this study. The paper search was not restricted to the intervention type, number of participants, origin of study, duration of the intervention, and/or follow-up. The exclusion criteria included adolescents and children, male adults, and patients with diseases (either associated with obesity development, such as T2D, cardiovascular disease, metabolic syndrome, or not associated with obesity). Single case reports, expert opinion manuscripts, letters to the editor, commentaries, conference papers, and review papers were excluded. Articles written in a language other than English were also excluded.

### 4.3. Study Selection

To assess studies and manage the study selection process, the web-based systematic review tool Covidence was used. Two reviewers (A.L and A.D) assessed independently uploaded publications. To eliminate duplicate publications, the initial evaluation was conducted using the EndNote 20 software and visual inspection for further confirmation. The screening included assessing the titles and abstracts based on the inclusion criteria. Then, studies accepted in the previous step were reviewed with full-text analysis. To ensure consensus on study selection, after each screening step, the decisions were made.

### 4.4. Risk of Bias Assessment and Evidence Quality Grading

The risk of bias assessment was performed by two reviewers. Quality assessment of selected peer-reviewed studies was prepared in accordance with the Cochrane collaboration’s risk of bias tool. Bias was categorized into seven domains (random sequence generation, inclusion and exclusion criteria, study design, blinding of outcome, performance, reporting, and other) and assessed into three groups of judgement (moderate, low, or no information). To visualize risk of bias assessments results, the robvis ROBINS-I tool was used [58].

### 4.5. Data Extraction

Data extraction was conducted by one reviewer (AL) following the Cochrane Public Health Group Data Extraction and Assessment Template. All relevant information from the manuscripts, e.g., treatment, sample size, participants’ characteristics (sex distribution, mean age), intervention (follow-up duration, type of collected tissue, study design, main results), and country where the intervention took place, was extracted. The bioinformatic analysis was conducted using supplementary data files, which contained the information about methylation values of samples analyzed pre- and post-surgery, CpG sites, and their location. The collected results were grouped into four time frames that were tissue-specific: 3 months, 6 months, 9–31 months, and 2 years. For further analysis and visualization, only female participants’ data who significantly lost weight after metabolic/bariatric surgery (*p* < 0.001) were used.

### 4.6. Data Quality Control

The supplementary data underwent quality control, and the CpG sites and their location were confirmed using EWAS Catalog Beta (https://www.ewascatalog.org/, accessed on 5 February 2023). The included studies were peer-reviewed with conducted statistical analyses and corrections, including the Benjamini–Hochberg [59] or Bonferroni adjustment [60]. Therefore, no additional statistical analyses were conducted for pre- and post-metabolic/bariatric surgery data before conducting pathway enrichment analysis.

### 4.7. KEGG Enrichment Analysis

Differentially methylated CpG sites from all publications were screened using the Kyoto Encyclopaedia of Genes and Genomes (KEGG) pathway analysis (https://www.kegg.jp, accessed on 10 October 2023). CpG sites with the highest expression changes (up- or down-regulated) were highlighted among the pathways where gene enrichment was the highest throughout each dataset. Grouped methylation changes based on the intervention timeline were visualized using generated heat maps and the pheatmap package within R 4.2.1. The highlighted pathways were further screened to determine their specific function and relevance to obesity or obesity-related comorbidities. Due to high number of highlighted pathways, the further analysis was focused on seven pathways associated with obesity or metabolic/bariatric surgery outcomes: forkhead box O (FoxO), Toll-like receptor, mammalian target of rapamycin (mTOR), cyclic AMP (cAMP), phosphatidylinositol 3-kinase/protein kinase B (PI3K-Akt), insulin and adipocytokine signaling pathway. Then, genes associated with obesity were further screened to determine their functions within this pathway and how the methylation changes after surgery affected their expression and function. The results of the highest methylation changes within different time frames were visually represented by the dot plot generated with Tableau 2024.1.

### 4.8. Differentially Methylated Loci Among Analyzed Tissues

To determine if there was a specific CpG site expressed in all tissue types, all obtained results were pooled together and searched with the R if there were any alignments present. Then the obtained results were visually presented using the Venn diagram. For seven selected pathways, the same search was conducted and visualized with a separate Venn diagram.

### 4.9. In-Silico Identification of DNA Sequence Variation Derived from the Epigenomic Meta-Analysis

Based on the methylation changes within selected pathways, obtained from pre- and post-metabolic/bariatric surgery epigenomic meta-analysis, a search for the single-nucleotide polymorphism (SNPs) was conducted using the GoDMC Database (http://www.mqtldb.org/, accessed on 3 November 2023) [61]. The data from these CpGs and associated SNPs will provide information on genetic variants that affect DNA methylation levels at CpG sites. The methylation quantitative trait loci analysis performed in this paper further supports the existing literature on CpGs in successful metabolic/bariatric surgery patients.

## 5. Conclusions

The current epigenomic meta-analysis revealed associations between methylation changes and successful weight loss after metabolic/bariatric surgery. The DNA methylation changes were associated with adipocytokine, insulin, cAMP, PI3K-Akt, Toll-like receptor, and mTOR signaling pathways, which play critical roles in adipocyte differentiation, inflammation, insulin sensitivity, glucose homeostasis, and body weight regulation. The methylation percentage changes within CpG sites vary depending on the timepoint at which they are analyzed after metabolic/bariatric surgery. Altered gene methylation within specific pathways can therefore be revealed in a specific time frame and may not be present in all analyzed timepoints. This study highlights the molecular (epigenetic signals) that are detectable prior to weight loss surgery, which are associated with successful weight loss post-surgery, can be potentially used in prevention strategies for obesity and obesity-related disorders once the findings have been replicated. The analysis of pathway-specific methylation changes in three tissue types revealed that the *LPIN1* gene correlated with multi-tissue insulin resistance. The results from unbiased screening of all included EWAS revealed that the *ALDH1A3* gene is involved in adipocyte differentiation and insulin regulation. Therefore, it is recommended that those genes should be further studied as potential molecular signatures for successful weight loss after metabolic/bariatric surgery. To the knowledge of the authors, this is the first epigenomic analysis that links specific pathways related to metabolic recovery after weight loss in female participants.

## Figures and Tables

**Table 1:** The summary of studies analyzing the effect of metabolic/bariatric surgery on DNA methylation.

Ref.	Population Age (y)	Number of Participants (Females/All)	Tissue	Type of Intervention	Duration of Data Collection	Study Design	Statistical Analysis	Main Results	Type of Study	Country
Day et al. (2017) [14]	33–59	7/7 (100%)	Muscle	Roux-en-Y gastric bypass (RYGB)	before and 3 months after the intervention	Reduced-representation bisulfite sequencing (RRBS), Illumina HiSeq 2000, pyrosequencing (PyroMark Q96)	A paired Student t test. Pearson correlation analysis	The analysis of *SORBS3* revealed 30 CpG hypomethylated sites	Candidate genes	USA
Garcia et al. (2021) [15]	45.1 ± 3.6	7/7 (100%)	Muscle	Roux-en-Y gastric bypass (RYGB)	before and 3 months after the intervention	Reduced-representation bisulfite sequencing (RRBS), Illumina HiSeq 2000	A paired Student’s *t*-test and an unpaired Student’s *t*-test. Wilcoxon signed-rank and Mann–Whitney *U* test	45 CpG sites were hypomethylated, and 28 CpG sites were hypermethylated	Epigenome-wide association studies (EWAS)	USA
Nicoletti et al. (2016) [16]	35.5 ± 10.1	14/14 (100%)	Blood (buffy coats)	Roux-en-Y gastric bypass (RYGB)	before and 6 months after the intervention	Bisulfite conversion; 7900HT Fast Real-Time PCR System	A paired Student t test; Benjamini–Hochberg false discovery rate correction. Pearson’s correlations	Four CpG sites within *LINE1*, *5hmC*, *SERPINE-1* and *IL6*	Candidate genes	Brazil
Wolf et al. (2022) [17]	36.9 ± 10.2	24/24 (100%)	Blood (buffy coats)	Roux-en-Y gastric bypass (RYGB)	before and 6 months after the intervention	Infinium Human Methylation 450K Bead Chip (Illumina)	Shapiro–Wilk test, paired Student’s *t*-test, Pearson’s correlation analysis	Four CpG sites within three genes: two CpG sites within *AGRP*, one CpG site within *GHRL* and *POMC*	Candidate genes	Brazil
Benton et al. (2015) [18]	44 ± 10	15/15 (100%)	Intra-operative subcutaneous and omentum adipose tissue	Roux-en-Y gastric bypass (RYGB)	before and 9–31 months after the intervention (average 17.6 months)	Infinium Human Methylation 450K Bead Chip (Illumina)	A paired *t*-test, Pearson’s correlation, Benjamini–Hochberg, Bonferroni correction	3601 CpG sites in SC showed significant differential methylation, within 1889 annotated loci and intergenic regions	Epigenome-wide association studies (EWAS)	New Zealand
Andersson et al. (2022) [12]	44 ± 10	22/22 (100%)	Subcutaneous Adipocytes	Roux-en-Y gastric bypass	before, 2, 5, and 10 years after the intervention	Infinium Human Methylation 450K BeadChip (Illumina)	A paired *t*-test, false discovery rate (FDR), 5% was used as a significance threshold unless otherwise stated. A	7729 differentially methylated CpG sites (DMS) at 2 years showed no sign of return to baseline.	Epigenome-wide association studies (EWAS)	Sweden

**Table 2:** The list of selected SNPs associated with obesity from the methylation quantitative trait loci analysis.

Gene	Genetic Variant	Function	Alleles	Position	Reference
POMC	rs1042571	Most significantly associated with a higher weight loss after RYGB	A>G	chr2:25383887	[26]
POMC	rs934778	Has been reported as a risk factor that affects insulin sensitivity	G>A	chr2:25389224	[27]
GHRL	rs27647	Associated with weight control and obesity; C/C genotype of the growth hormone secretagogue receptor gene experienced most weight loss at 30 months	C>T	chr3:10332468	[28]
LEPR	rs1137101	Linked to a higher risk of T2D in patients with obesity; improved weight loss for the A/A genotype compared to homozygous carriers of the G allele at 12 and 24 months after RYGB	G>A	chr1:66058513	[29,30]
LEPR	rs1137100	Could be involved in the development of morbid obesity	G>A	chr1:66036441	[26]
LEP	rs7799039	Associated with obesity	A>G	chr7:127878783	[31]

**Table 3:** PICOS framework used to establish eligibility criteria.

PICOS Element	Criteria
Population	Female non-pregnant adults (18 years or older) with obesity and no underlying health conditions or diseases
Intervention	Bariatric surgery (any type)
Comparison	Pre- to post- data
Outcome	DNA hypermethylation or hypomethylation in CpG sites
Study design	Epigenomic-wide association studies and candidate genes studies

## Data Availability

The datasets used and/or analyzed during the current study are available from the corresponding author on reasonable request.

## Supplementary Materials

The following supporting information can be downloaded at: https://www.mdpi.com/article/10.3390/epigenomes9030032/s1, Supplementary File S1. The results of KEGG pathways analysis of the differentially methylated genes 31 pathways associated with obesity and weight loss. Supplementary File S2. A list of single-nucleotide polymorphisms (SNPs) revealed from seven highlighted pathways associated with obesity. Supplementary File S3. The details of 28 genes that were differentially methylated in both adipose tissue and adipocytes from EWAS results displayed in Figure 6. Supplementary File S4. A list of all genes revealed during data preparation for a Venn diagram.

## Abbreviations

The following abbreviations are used in this manuscript:

5hmC	5-Hydroxymethylcytosine
ADAP1	ArfGAP with dual PH domains 1
ARGP	agouti-related neuropeptide gene
AKT1	AKT serine/threonine kinase 1
AKT2	AKT serine/threonine kinase 2
ALDH1A3	aldehyde dehydrogenase 1 family member A3
BCL2	BCL2 apoptosis regulator
C2CD4B	C2 calcium-dependent domain-containing 4B
cAMP	cyclic AMP
CFAP44	cilia and flagella-associated protein 44
COL4A1	collagen type IV alpha 1 chain
DNAJC5G	DnaJ heat shock protein family member C5 gamma
EMX2OS	opposite strand/antisense RNA
EWAS	epigenome-wide association studies
EWL	excess weight loss
FHOD3	formin homology 2 domain-containing 3
FoxO	forkhead box O
FOXO1	Forkhead box O1
GHRL	Ghrelin
IL6	interleukin 6
KEGG	Kyoto Encyclopaedia of Genes and Genomes pathway analysis
LEP	Leptin
LEPR	Leptin receptor
LINE1	long interspersed element-1
LPIN1	Lipin 1
LRP5	LDL receptor-related protein 5
MAGEL2	MAGE family member L2
MAPK10	mitogen-activated protein kinase 10
MEDLINE	Medical Literature Analysis and Retrieval System Online
mTOR	mammalian target of rapamycin
NHS	National Health Service
OBSCN	obscurin, cytoskeletal calmodulin, and titin-interacting RhoGEF
OSMR	oncostatin M receptor
OVAT	visceral adipose tissue
PABPC1	poly(A) binding protein cytoplasmic 1
PI3K-Akt	phosphatidylinositol 3-kinase/protein kinase B
PIK3R1	phosphoinositide-3-kinase regulatory subunit 1
PIK3R2	phosphoinositide-3-kinase regulatory subunit 2
PITX2	paired like homeodomain 2
PKN2	protein kinase N2
POMC	Proopiomelanocortin
PRISMA-P	Preferred Reporting Items for Systematic Review and Meta-Analysis Protocols
PRKAB2	protein kinase AMP-activated non-catalytic subunit beta 2
PRKAG2	protein kinase AMP-activated non-catalytic subunit gamma 2
RRBS	reduced-representation bisulfite sequencing
SAT	subcutaneous adipose tissue
SERPINE-1	serpin family E member 1
SGK1	serum/glucocorticoid-regulated kinase 1 gene
SIM1	SIM bHLH transcription factor 1
SNPs	single-nucleotide polymorphisms
SORBS3	sorbin and SH3 domain-containing 3 gene
T2D	type 2 diabetes
TLR1	Toll-like receptor 1
TRAF6	TNF receptor-associated factor 6
VEGFC	vascular endothelial growth factor C
Wnt10b	Wnt family member 10B
Wnt5b	Wnt family member 5B
